# Guided Self-Assembly of ES-Derived Lung Progenitors into Biomimetic Tube Structures That Impact Cell Differentiation

**DOI:** 10.3390/bioengineering8120209

**Published:** 2021-12-10

**Authors:** John P. Soleas, Linwen Huang, Elisa D’Arcangelo, Maria Cristina Nostro, Thomas K. Waddell, Alison P. McGuigan, Golnaz Karoubi

**Affiliations:** 1 Institute for Biomaterials and Biomedical Engineering, University of Toronto, 164 College Street, Toronto, ON M5S 3G9, Canada; john.soleas@mail.utoronto.ca (J.P.S.); linwenh.huang@mail.utoronto.ca (L.H.); elisa.darcangelo@uhnresearch.ca (E.D.); tom.waddell@uhn.ca (T.K.W.); 2 Latner Thoracic Surgery Research Laboratories, Toronto General Hospital, 101 College Street, Toronto, ON M5G 1L7, Canada; 3 McEwen Stem Cell Institute, University Health Network, 101 College Street, Toronto, ON M5G 1L7, Canada; Cristina.Nostro@uhnresearch.ca; 4 Department of Physiology, University of Toronto, 1 King’s College Circle, Toronto, ON M5S 1A8, Canada; 5 Institute of Medical Science, University of Toronto, 1 King’s College Circle, Toronto, ON M5S 1A8, Canada; 6 Department of Chemical Engineering and Applied Chemistry, University of Toronto, 200 College Street, Toronto, ON M5S 3E5, Canada; 7 Department of Mechanical and Industrial Engineering, University of Toronto, 5 King’s College Rd., Toronto, ON M5S 3G8, Canada; 8 Department of Laboratory Medicine and Pathobiology, 1 King’s College Circle, Toronto, ON M5S 1A8, Canada

**Keywords:** pluripotent stem cells, lung progenitor cells, geometric confinement, mechanical cue, biomaterial, extracellular matrix, directed differentiation

## Abstract

Chemically directed differentiation of pluripotent stem cells (PSCs) into defined cell types is a potent strategy for creating regenerative tissue models and cell therapies. In vitro observations suggest that physical cues can augment directed differentiation. We recently demonstrated that confining human PSC-derived lung progenitor cells in a tube with a diameter that mimics those observed during lung development results in the alteration of cell differentiation towards SOX2^−^SOX9^+^ lung cells. Here we set out to assess the robustness of this geometric confinement effect with respect to different culture parameters in order to explore the corresponding changes in cell morphometry and determine the feasibility of using such an approach to enhance directed differentiation protocols. Culture of progenitor cells in polydimethylsiloxane (PDMS) tubes reliably induced self-organization into tube structures and was insensitive to a variety of extracellular matrix coatings. Cellular morphology and differentiation status were found to be sensitive to the diameter of tube cells that were cultured within but not to seeding density. These data suggest that geometric cues impose constraints on cells, homogenize cellular morphology, and influence fate status.

## 1. Introduction

Directed differentiation of human embryonic stem cells and induced pluripotent stem cells into defined cell types offers the opportunity for regenerative cell therapy and in vitro disease modelling for novel target and therapy discovery. The gold standard to create large cell numbers of human progenitors for such models is directed differentiation from human pluripotent stem cell (PSC) populations. These directed differentiation protocols are capable of recapitulating much of the diversity within a target organ by exposing the PSCs to a specific sequence of chemical signals designed to mimic developmental cues characterized from in vivo human and mouse studies [1,2,3,4,5,6,7,8,9,10,11]. While this approach has enabled great progress, in many tissues, challenges remain, such as the poor yield of specific cell populations, variability in the proportion of each cell population produced, and a lack of maturity beyond fetal levels. A need, therefore, exists to further enhance our capabilities to control the fate and heterogeneity of the cell populations produced using these protocols.

Current directed differentiation strategies have focused extensively on replicating the chemical signals that occur during lung specification in vivo. Differentiating cells, however, also experience a number of physical cues associated with morphogenetic changes in developing tissues [12,13,14] that currently are not typically replicated in standard differentiation protocols. For example, during lung organogenesis, the simple anterior foregut endoderm tube branches into the complex arborized structure of the adult lung through a highly stereotyped mechanism of branching morphogenesis which facilitates the outgrowth, placement, and structure of all lung airways [12]. During this branching process, epithelial cells of the lung self-organize into a series of transient structures with defined architectural motifs. The process of morphogenesis in the lung occurs in concert with the differentiation of the developing epithelium, and proximal markers, such as those found in the cells of the trachea, precede those found more distally in the putative alveolus [15,16]. Interestingly, manipulating the shape of developing lung tubes in vivo was found to impact cell fate [17,18], suggesting physical cues have the potential to impact cell differentiation. Furthermore, in vitro, a number of observations in the literature suggest that physical cues can provide a potential strategy to augment directed differentiation: manipulation of stiffness through cell-to-cell and cell-to-matrix interactions has been shown to increase the proportion of cells that express endoderm markers [19]; sub-cellular curvature has been shown to alter mesenchymal stem cell differentiation towards osteogenic or adipogenic fate in equipotential media [20]; shear stress has been shown to direct primed ES lung progenitors towards a type I alveolar epithelial cell [21]; utilizing magnetic beads to create mechanical stretch, distal lung progenitors have been found to become type II airway epithelial cells (AEC II) [22,23]; finally, patterned 3D geometry has been shown to alter the organization of heterogenous tissues, with specific cell types sorting to the cell–matrix boundary [24], and to alter the branching morphogenesis of mammary tissue [25]. Taken together, these examples suggest that controlling the physical cues experienced by a differentiating cell population is a potential strategy to direct cell fate.

In all the examples described above, except for fluid-based shear stress, the physical cues did not replicate those experienced physiologically during development. Recently, we demonstrated that confining human ES-derived lung progenitor cells into a tube with dimensions selected to specifically mimic structures observed during lung development results in an alteration in cell fate and a bias towards the production of distal lung cells [26]. Specifically, we showed that bipotent and dual positive (SOX2^+^SOX9^+^) progenitor cells became SOX9^+^ over three days when cultured in 100 µm tubes as opposed to on flat substrates. Furthermore, upon removal from the tubes and exposure to further chemical differentiation, cells from flat cultures were capable of producing both proximal and distal epithelia, while cells retrieved from tubes were only capable of forming distal epithelia. This phenomenon was observed when cells were cultured in both tubes and on posts with the appropriate diameter. The mechanism of this change in differentiation behavior was found to be a mechanobiological effect dependent on canonical Wnt signaling and tension. Here we set out to assess the robustness of this geometric confinement effect with respect to different culture parameters to determine the feasibility of using such an approach to enhance directed differentiation protocols. Specifically, we set out to test the impact of extracellular matrix coating composition and cell seeding density of ES-derived lung progenitors (NKX2.1^+^FOXA2^+^SOX2^+^SOX9^+^) on monolayer formation within tubular constructs, explore the corresponding changes in cell morphometry, and finally assess the sensitivity of the change in SOX2–SOX9 status to these culture parameters.

## 2. Material and Methods

### 2.1. PDMS Device Manufacture

**Figure 1:**
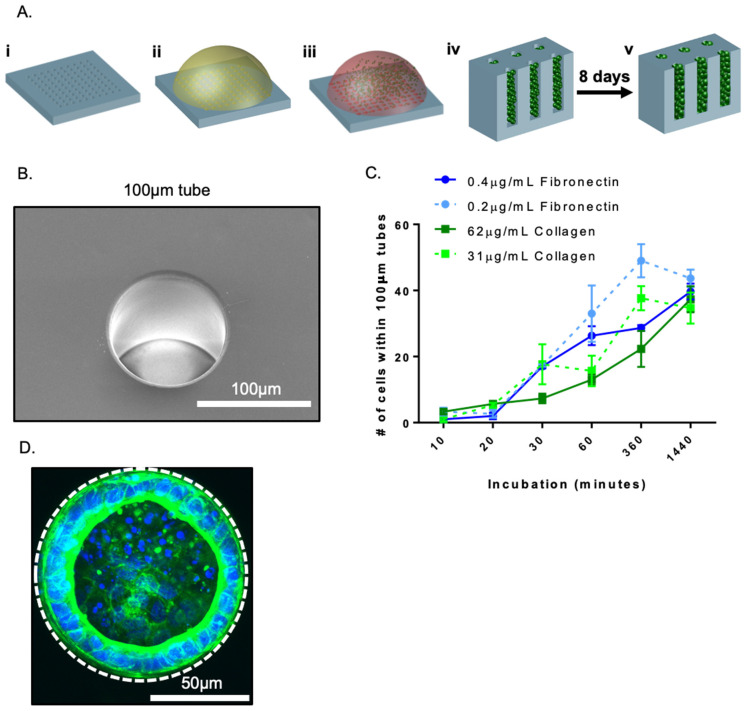
Schematic of tubular self-assembly. (**A**) PDMS substrates containing tubes of the desired dimensions were generated by moulding against a topographically patterned master produced using photolithography (**i**). Substrates were coated with an extracellular matrix overnight (**ii**) and subsequently were coated in day 17 progenitor cells at a selected density (**iii**) which were allowed to adhere for 25 min before non-adherent cells were washed off the substrate surface (**iv**); cells seeded into cavities self-organized into monolayers (**v**) within the architecture over a period of 3 to 8 days. (**B**) Representative scanning electron microscopy of 100 µm tubes; bar, 100 µm. (**C**) Quantification of the number of cells within 100 µm tubes as a function of incubation time; n = 3. (**D**) Representative fluorescent micrograph of day 17 lung progenitor cells, F-actin (green) and nuclei (blue), after 2 days of culture seeded within 100 µm tubes; bar, 50 µm; the dotted white line approximates the PDMS border.

The manufacture of our PDMS devices is shown in schematic form in Figure 1A. Silicon masters with post diameters of 100 and 400 µm for polydimethylsiloxane (PDMS) stamp generation were fabricated using standard photolithography techniques, as described previously [27]. These dimensions were selected based on in vivo tube size at a relevant embryonic stage and our previous studies, where the differentiation effect was not present in 400 µm tubes. Briefly, PDMS moulds were created by pouring liquid and degassed PDMS polymer (10:1 *w/w* base:crosslinker) onto our silicon masters and baking them in an oven at 68 °C overnight. After baking, the PDMS moulds were allowed to cool before subsequent removal from our silicon wafer master (Figure 1A). Moulds were then individually cut and liquid PDMS was used to adhere them to a 24-well plate (Millipore Sigma, Ontario, Canada). These PDMS devices were washed 24 h prior to cell seeding with 70% ethanol and allowed to dry in a bio-safety cabinet while under exposure to UV light for 30 min.

### 2.2. Scanning Electron Microscopy

Tube moulds of 100 and 400 µm were mounted onto electron microscopy stubs (Electron Microscopy Sciences, Hatfield, PA, USA). Sample were gold coated using a Polaron PS3 system (Lewes, UK) and imaged with a Hitachi SU 3500 VPSEM (Hitachi High Technologies Canada, Toronto, ON, Canada) at 5 kV.

### 2.3. Human Embryonic Stem Cell Culture

Briefly, hES3 human embroynic stem cells were maintained on mitomycin C, growth inhibited mouse embryonic feeders seeded at a density of 60,000 cells/cm^2^ (Nagy Laboratory, Lunenfeld Research Institute). hES were cultured in DMEM/F12 (Thermo Fisher Scientific, Mississauga, ON, Canada) supplemented with 20% (*v/v*) KnockOut serum, 1× non-essential amino acids, 1× glutamine; 1×x antibiotic–antimycotic (Thermo Fisher Scientific); 50 mM 2-mercaptoethanol (Millipore-Sigma, Oakville, ON, Canada); 20 ng/mL FGF2 (R&D Systems, Oakville, ON, Canada).

Media was changed daily, and cells were passaged using TrypLE (every 5 days at a 1:6 ratio) and maintained in ROCK inhibitor (10 µM Y27632 (Tocris, Oakville, ON, Canada)). Cultures were maintained in a 5% CO_2_ air environment at 37 °C.

### 2.4. Lung Directed Differentiation

Lung progenitors were differentiated following previously published protocols [6,28]. Briefly, hES3 (gifted by Stephen Elefanty, Monash University) were MEF depleted on matrigel for 24 h and then passaged used TrypLE. Following passaging, hES3 were seeded into primitive streak-inducing media in ultra-low attachment plates for 24 h to create embryoid bodies. This was followed by 4 days of definitive endoderm (DE) inducing media (Activin A, BMP4, FGF2, and Y27632). A complete media exchange was performed on day 3 with fresh DE media. Following DE induction, cells were dissociated using 0.25% trypsin (Corning) for 5 min and quenched with an equal volume of fetal bovine serum (Thermo Fisher Scientific). Cells were then collected, counted, and seeded onto 0.2% coated fibronectin 6-well plates with anteriorization media 1 (SB431542 and dorsomorphin) for 48 h, followed by anteriorization media 2 (SB431542 and IWP2) for 48 h. Cell media was then changed to ventralization media (FGF10 and 7, BMP4, CHIR 99021, and all-trans retinoic acid) for 10 days, with complete media changes occurring every other day. All growth factors were from R&D Systems, and small molecules inhibitors were from Tocris.

### 2.5. Extracellular Matrix Screening

A commercially available extracellular matrix (ECM) screening plate from Advanced Biomatrix (San Diego, CA, USA) was used according to the manufacturer’s protocol. Briefly, lung progenitor cells at day 17 were passaged and seeded onto the plate for 3 days with a single media change on day 2.

### 2.6. Cell Seeding onto PDMS Architecture

Prior to cell seeding, PDMS moulds were washed with phosphate buffered saline (PBS) (Thermo Fisher) and coated with one of the following mixtures: 125 µg fibronectin (Millipore-Sigma, Oakville, ON, Canada) and 125 µg/mL PurCol (Col I) (Advanced Biomatrix, San Diego, CA, USA), all dissolved in a 0.01 N hydrochloric acid solution; or 125 µg fibronectin, 125 µg vitronectin (Advanced Biomatrix); or 125 µg fibronectin, 125 µg collagen IV (Advanced Biomatrix).

All mixtures were kept on moulds overnight at 37 °C. Just prior to cell seeding the PDMS inserts were washed with phosphate buffered saline (PBS) (Thermo Fisher Scientific, Burlington, ON, Canada). Following ECM coating, day 17 lung progenitors were passaged and counted using a haemocytometer and seeded at a given cell number in 130 µL of ventralizing media. After initial seeding, cells were allowed to adhere for 25 min and subsequently washed with warmed PBS. Media was exchanged every other day for the duration of culture.

### 2.7. Immunocytochemistry 

Immunocytochemistry was performed by fixing samples with 4% paraformaldehyde (Thermo Fisher Scientific) for 10 min at room temperature. Following fixation, samples were washed 3 times with PBS, permeabilized with 0.1% Triton X-100 (Millipore-Sigma) for 20 min at room temperature, washed with PBS, and then blocked with a 3% bovine serum albumin (BSA, Millipore-Sigma) solution for 30 min. Samples were then stained with selected antibodies, such as 4′,6-diamidino-2-phenylindole (DAPI 1:300; Millipore Sigma, D9542-1MG) and tetramethylrhodamine (TRITC-phalloidin 1:300; Millipore Sigma, P1951-.1MG), or SOX2 (1:200, Thermo Fisher, MA1-014), SOX9 (1:1000, Millipore Sigma, HPA001758) and Beta-catenin (1:100, Cell Signaling Technologies, 8480), overnight at 4 °C. Following overnight staining, samples were washed with 3% BSA three times and then stained with one of the following secondary antibodies at a dilution of 1:250 where appropriate: Goat anti-Rabbit IgG (H + L) Secondary Antibody, Alexa Fluor^®^ 633 conjugate (Thermo Fisher, A-21070) and Goat anti-Mouse IgG (H + L) Cross-Adsorbed Secondary Antibody, Alexa Fluor 633 (Thermo Fisher, A-21050), and Goat anti-Rabbit IgG (H + L) Cross-Adsorbed Secondary Antibody, Alexa Fluor 405 (Thermo Fisher, A-31556). Samples were then washed again with 3% BSA three times and kept in 3% BSA until they were imaged on an Olympus IX81 inverted confocal microscope (Olympus Canada Inc., Toronto, Canada); as samples were hundreds of microns thick, samples were imaged with a 40×/0.8 NA LUMPlanFI dipping lens (water) using z-stacks with 5 µm optical slices. 

### 2.8. Quantification of Cell Seeding Area and Cell Metrics 

Cell dimensions, such as cell radius, area, and perimeter, were analyzed using Cell Profiler. Images were converted to greyscale, cropped, and then objects were manually traced. Metrics were quantified using an algorithm, conversion factors applied, and the results averaged.

### 2.9. Statistics

One-way analysis of variance (ANOVA) or multiple Student’s *t*-tests with a Holm–Sidak correction were used to test for significant differences among multiple test groups. Equal variance was assessed using the Brown–Forsythe test. Significant differences from one-way ANOVA were identified using the Tukey post hoc test. When variance was unequal a Greenhouse–Geisser correction was applied. Results are shown as mean ± standard error of the mean (SEM); *p*-values < 0.05 were considered statistically significant. N refers to independent biological replicates. All statistical analysis was performed in GraphPad (GraphPad Software V6, La Jolla, CA, USA).

### 2.10. Ethical Considerations

All human pluripotent stem cell line usage was in accordance with the guidelines of the Stem Cell Oversight Committee (SCOC) of the Canadian Institute of Health Research (CIHR), who approved the current project in March 2015.

## 3. Results

### 3.1. Culture of Progenitor Cells in Replica Moulded PDMS Tubes Reliably Induced Self-Organization into Tube Structures

During the stage of lung development when proximal and distal epithelial cells are specified, the lung consists of a series of tubes with dimensions in range of 25–100 microns in diameter [29,30,31]. To mimic the architecture and scale of these structures we exploited soft photolithography [32,33]. Polydimethylsiloxane (PDMS) substrates containing blind-ended tubes with diameters of 100 µm were created using photolithography and replica moulding. Scanning electron microscopy confirmed the expected tube diameter (Figure 1B) and optical microscopy of tube profiles showed that tubes had a depth of 127 ± 11.2 µm (mean ± standard error of the mean (SEM)). After fabrication, substrates were coated with collagen or fibronectin, typical extracellular coatings used for air–liquid interface (ALI) culture and in previous differentiation protocols [6,28,34], respectively. Substrates were then seeded with day 17 lung progenitor cells (a population that we and others previously demonstrated is bipotent [5,6]) and incubated to allow adhesion of the cells to the tube surface. To balance the seeding of the cells within the tubes, as opposed to on the top surface of the PDMS, we varied the incubation time on the substrate before washing (Figure 1C). As expected, too low an incubation time before washing resulted in a scarcity of cells within tubes and many tubes containing no cells. If an insufficient number of cells were contained within a tube, cell death occurred within 48 h. Conversely, long incubation times prior to washing resulted in aggregate formation within some tubes. Similar results were obtained for a range of collagen and fibronectin concentrations. For long incubation times however, if the substrate was washed, by pipetting PBS over the seeded substrate using a sufficient volume of wash solution, these aggregates were dislodged and aspirated from the tubes. Therefore, above a critical incubation time, similar numbers of cells could be seeded within each tube using our simple incubation method, resulting in the formation of a continuous monolayer lining the PDMS tubes over a period of 48 h (Figure 1D).

### 3.2. Epithelial Tube Formation Was Insensitive to Substrate Extracellular Matrix Coating Composition

**Figure 2:**
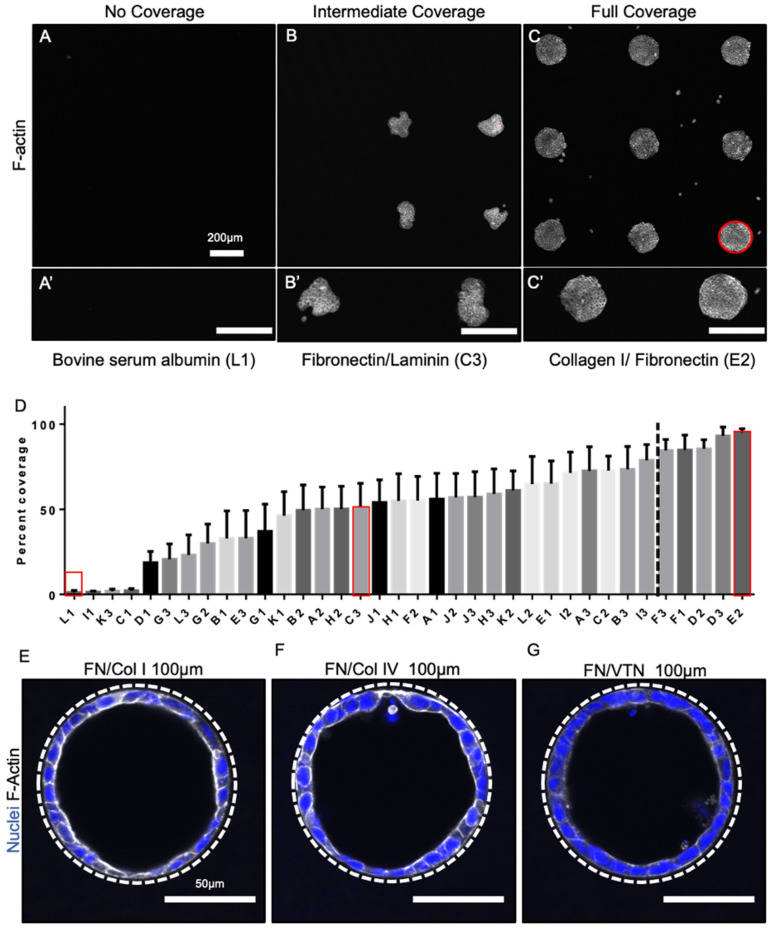
Selection of extracellular matrix coating for tubular constructs. Example F-actin (white) fluorescent micrographs of no coverage (**A**), intermediate coverage (**B**), and full coverage (**C**) with corresponding zoomed-in micrographs (**A’**–**C’**); bar 200 µm. (**D**) Bar chart showing percent coverage with red outlines of the above conditions; the dotted line corresponds to 80% percent coverage. Fluorescent micrographs of day 17 lung progenitors seeded in tubes for 3 days and stained with DAPI (blue) and F-actin (white) from selected ECM coatings showed that fibronectin and collagen I (**E**), fibronectin and collagen IV (**F**), and fibronectin and vitronectin (**G**) seeded 100 µm constructs formed monolayers. Bar, 50 µm; the dotted white line approximates the PDMS border; n = 3.

Having determined that incubating the cells in the tubes for approximately more than 20 min enabled monolayer formation in 100-micron tubes we next set out to determine if monolayer formation was sensitive to the use of different extracellular matrix coatings on the substrate surface. To do this we first assessed the adhesion of day 17 lung progenitors to a variety of standard extracellular matrix coatings using a commercially available matrix screening array from Advanced BioMatrix. On this array, various extracellular matrix proteins are patterned in a 3 × 12 array of various combinations. A high-density collagen (250 µg/mL) positive control and BSA non-adhesive negative control were also included (ECM composition array details are shown in Supplementary Figure S1). Within each of these grids, each extracellular matrix protein is plotted in a 3 × 3 setup. Day 17 lung progenitor cells were seeded on the array and allowed to adhere and spread for 3 days, then coverage was quantified using fluorescence microscopy of F-actin staining. The percent of cell coverage was then calculated using the known area of the ECM spot specified by the manufacturer. Examples of no, intermediate, and full coverage spots are shown in Figure 2A–C. Zoomed-in micrographs in Figure 2A’–C’ also show the range of cell morphologies (spreading) observed (F-actin; white). The highest degrees of cell adhesion (80%+) were observed on spots containing a combination of extracellular matrix proteins (Figure 2D), as opposed to single protein coatings. The combination with the best percentage of coverage was collagen I and fibronectin (94.9 ± 2.3%), followed closely by fibronectin and vitronectin (93.1 ± 5.1%).

To then assess the effect of ECM composition on the ability of day 17 progenitor cells to form and maintain a continuous monolayer in the PDMS tubes for 72 h we seeded substrates coated with the top two compositions and an intermediate composition (specifically, fibronectin and vitronectin (achieved 93.1 ± 5.1% coverage); collagen IV and fibronectin (achieved 72.5 ± 14.2% coverage)). Fluorescent micrographs, shown in Figure 2E–G, show that all three ECM combination resulted in completely cellularized tubes with a continuous epithelial monolayer (Figure 2E–G). This suggested that given cells are incubated in the tubes for enough time, that a sufficient number adhere to maintain viability and form a continuous monolayer, and that the specific adhesive properties of the tube are not a critical factor. We speculate that, once within the tube, the cells secrete additional matrix to sustain prolonged growth and attachment.

### 3.3. Fate Changes in 100 µm Tubes Were Insensitive to Substrate Extracellular Matrix Coating Composition

**Figure 3:**
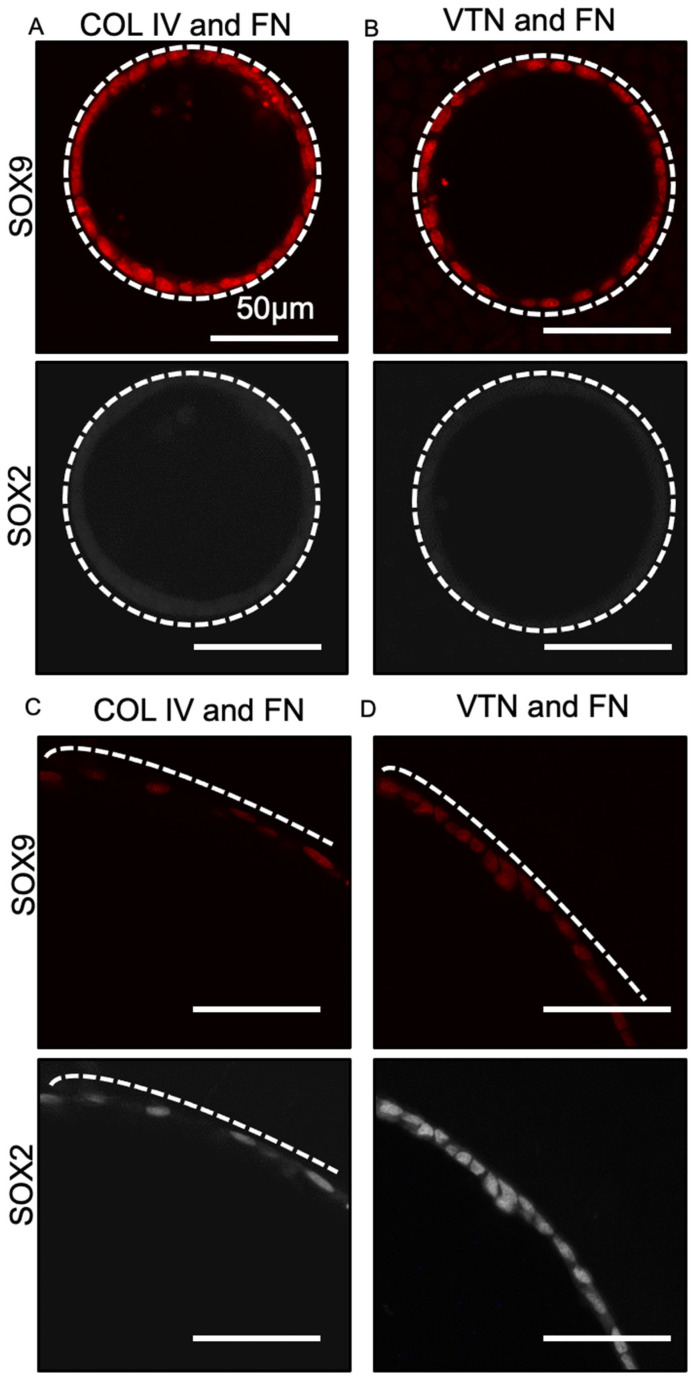
SOX2–SOX9 status of lung progenitors seeded in 100- and 400-micron tubes with different matrix coatings. Fluorescent micrographs of day 17 lung progenitors seeded for 8 days at 600,000 cells/cm^2^ collagen IV and fibronectin (**A**) and vitronectin and fibronectin (**B**) show that in 100 µm tubes the cells are SOX9+, while the cells in 400 µm (**C**,**D**) remain SOX2^+^SOX9^+^. Bar, 50 µm; the dotted white line approximates the PDMS border; n = 3.

Our previous work showed that day 17 progenitor cells cultured in tubes coated with fibronectin and collagen I resulted in loss of SOX2 expression in 100 µm but not 400 µm tubes. To confirm that this effect was still observed on the newly selected matrix coatings, we assessed the expression of SOX2 and SOX9 of lung progenitors after 8 days’ culture in 100 and 400 µm tubes. As expected, SOX2 expression was lost in progenitor cells cultured in 100 µm tubes coated with either collagen IV and fibronectin or vitronectin and fibronectin, while SOX9 continued to be expressed (Figure 3A,B). Progenitor cells seeded in 400 µm PDMS tubes coated with either collagen IV and fibronectin or vitronectin and fibronectin (Figure 3C,D, respectively) did not show loss of SOX2 expression and remained SOX2^+^SOX9^+^. This suggested that culture in tubes coated with all three ECM compositions identified in our screen produced the same effect on lung progenitor differentiation status. For future experiments, we therefore selected fibronectin and collagen I for several reasons: first, fibronectin and collagen I produced the highest percent coverage on the extracellular matrix protein screening array; second, standard directed differentiation and air–liquid interface protocols typically use fibronectin and collagen to facilitate cell adhesion [6] and maturation [35].

### 3.4. Progenitor Cells Self-Assemble into a Confluent Polarized Monolayer Lining the Tube Mould within 48 h of Seeding 

**Figure 4:**
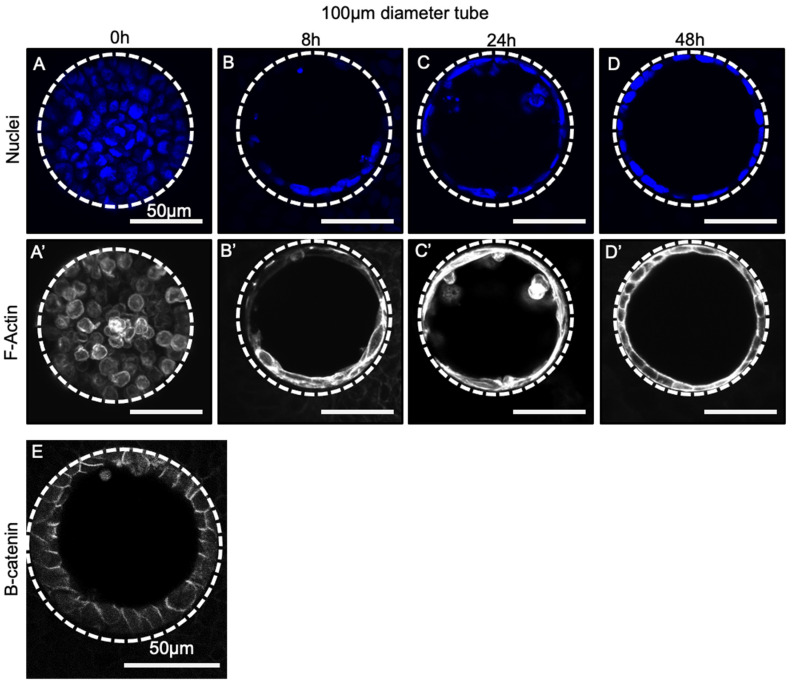
Tube formation dynamics over 48 h in 100 µm tubes. Fluorescent micrographs of 100 µm tubes seeded with day 17 lung progenitors and stained with DAPI (blue) at 0 h (**A**), 8 h (**B**), 24 h (**C**), and 48 h (**D**) showing partial tube formation by 8 h and complete formation occurring within 48 h. Corresponding F-actin staining (white) in (**A’**–**D’**) reveals a similar pattern. (**E**) Beta-catenin staining (white) was also assessed to characterize cell polarization within 100 µm tubular constructs. Bar, 50 µm; the dotted white line approximates the PDMS border; n = 3.

Having selected an appropriate ECM coating that robustly generated confluent monolayers within the PDMS tubes, we next sought to assess the dynamics of progenitor self-assembly within the tubes. To do this, we assessed cellular structures at various time points between 0 and 48 h after cell seeding. At 0 h (Figure 4A), the majority of cells appeared to be floating within the PDMS tube: nuclei (blue) were rounded and were not localized to the substrate walls and F-Actin staining (Figure 4A’, white) was diffuse, suggesting the cells had not adhered. By 8 h (Figure 4B,B’), partial coverage of the PDMS tube walls occurred and cells began to exhibit squamous morphology around the tube: nuclear staining co-localized with the PDMS walls, and elongated and cortical actin was visible. By 24 h (Figure 4C,C’) after seeding, the PDMS walls of the tube were mostly covered with a confluent epithelial layer with a squamous morphology; however, remnants of non-adherent round cells were still visible. These were washed away in subsequent media exchanges, however. By 48 h after cell seeding (Figure 4D), a confluent monolayer was visible, completely covering the PDMS tube walls with nuclei, and F-Actin staining (Figure 4D’) indicated the formation of a single-cell thick layer. As expected, the intensity of F-actin staining appeared to increase in both apical (lumen-facing) and basal (wall-facing) cell compartments. Furthermore, cells appeared to be polarized as indicated by observed enrichment of beta-catenin at the lateral membranes of cells lining the tubes (Figure 4E). Similar self-organization dynamics were also observed in larger diameter tubes of 400 µm (Supplementary Figure S2), suggesting that tube formation rate is similar regardless of tube size. We speculate that this is because as long as a critical density of cells are seeded within the tube (i.e., the incubation time before washing is long enough) the dynamics of tube formation are controlled by cell–cell and cell–substrate interactions, as opposed to macroscopic geometry.

### 3.5. Cellular Morphology Is Sensitive to Tube Diameter but Not Seeding Density

**Figure 5:**
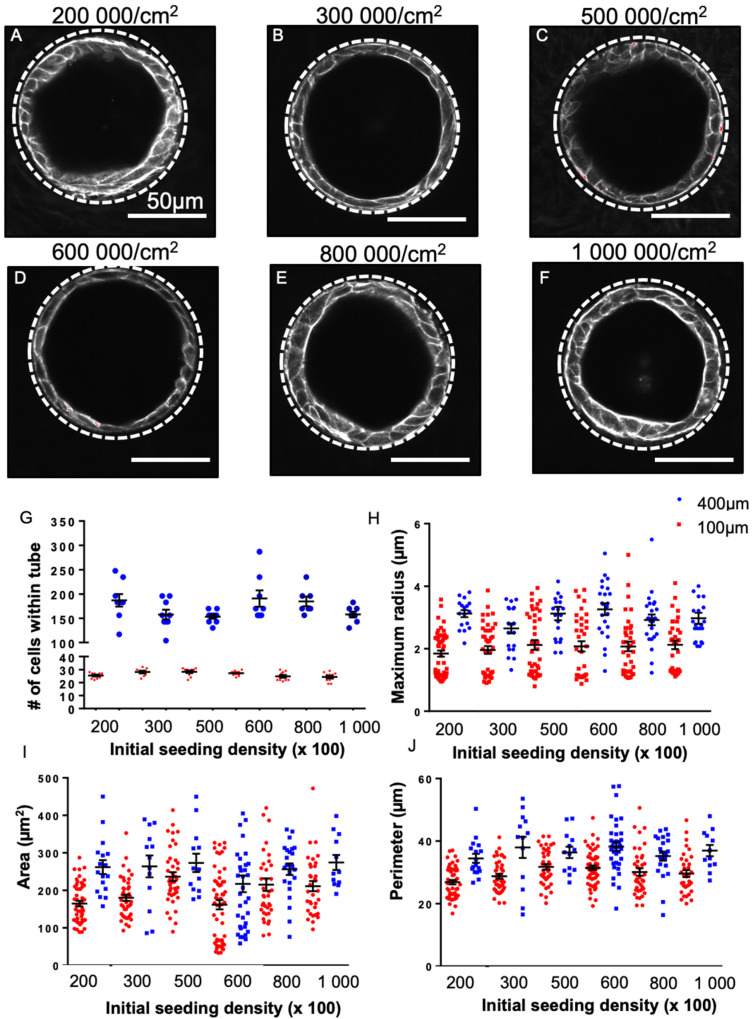
Cell seeding over a range of densities and corresponding cell morphometry. Fluorescent micrographs of day 17 lung progenitor cells seeded for 8 days in 100 µm tubes and stained for F-actin (white) at 200,000 cells/cm^2^ (**A**), 300,000 cells/cm^2^ (**B**), 500,000 cells/cm^2^ (**C**), 600,000 cells/cm^2^ (**D**), 800,000 cells/cm^2^ (**E**), 1,000,000 cells/cm^2^, (**F**) shows that tube formation occurs without aggregate formation at these cell seeding densities with appropriate washing. The number of cells per tube, 100 and 400 µm (**G**), was quantified; no significant differences were found between seeding conditions within the same sized tubes; n = 3. (**H**–**J**) morphometric analysis of 100 (red) and 400 µm (blue) tubes at various seeding densities. The maximum radius (**H**), area (**I**), and perimeter (**J**) of cells seeded in 400 µm tubes were consistently larger than cells seeded in 100 µm tubes. Bar, 50 µm; the dotted white line approximates the PDMS border.

Our previous work demonstrated that cells in 100-micron tubes experienced a greater deformation than in 400-micron tubes to conform to the curvature of the tube surface. We therefore hypothesized that the difference in SOX2 loss between 100- and 400-micron diameter tubes was due to differences in cell geometry [26]. We therefore set out here to better understand how the architecture of the epithelial monolayer within the PDMS tube differed between tubes of 100- and 400-micron diameter at day 8 after seeding and if the initial seeding density (given a sufficient incubation time before washing to achieve complete cell adhesion) impacted this architecture. We selected a wide range of cell seeding densities well above those used in initial experiments (200,000 cells/cm^2^; 300,000 cells/cm^2^; 500,000 cells/cm^2^; 600,000 cells/cm^2^; 800,000 cells/cm^2^; and 1,000,000 cells/cm^2^) and assessed changes in the structures formed. In all samples, cells formed a monolayer in the 100 µm tubes by 48 h, and by 8 days did not show any difference in gross F-actin staining (Figure 5A–F). Similar results were observed in 400 µm tubes (Supplemental Figure S3). To assess the geometry of the cells within the monolayer lining the differently sized tubes, we quantified the number of cells per tube and the shape of each cell using actin staining to define maximum radius (the largest width of the cell), perimeter, and area. The initial seeding density did not impact the number of cells contained in the monolayer lining the tube wall for a given tube diameter (Figure 5G); however, the number of cells within 100- versus 400-micron tubes was significantly different. Consistent with this, initial cell seeding density did not impact cell shape (for a given tube diameter): Tubes with the same diameter had similar maximum radii, areas, and perimeters (Figure 5 H–J). The size of the tube did, however, impact cell geometry: maximum radius was consistently and significantly (*p* < 0.05) larger in 400 µm tubes than in 100 µm tubes (Figure 5H): In 400 µm tubes, cells at all densities averaged 3.00 ± 0.09 µm, while in 100 µm tubes, cells at all densities averaged 2.03 ± 0.04 µm. Similarly, cell area (Figure 5I) and perimeter (Figure 5J) showed the same significant trend (*p* < 0.05). The larger cell spreading in 400 µm tubes is consistent with our previous observations that cell contractility in 400 µm tubes is lower than in 100 µm tubes, which should result in less cell compaction in 400 µm tubes. Overall, these data suggest that monolayer geometry at day 8 is insensitive to cell seeding density (after washing) but defined by tube diameter.

### 3.6. SOX2 Status Is Sensitive to Tube Diameter but Not Seeding Density

**Figure 6:**
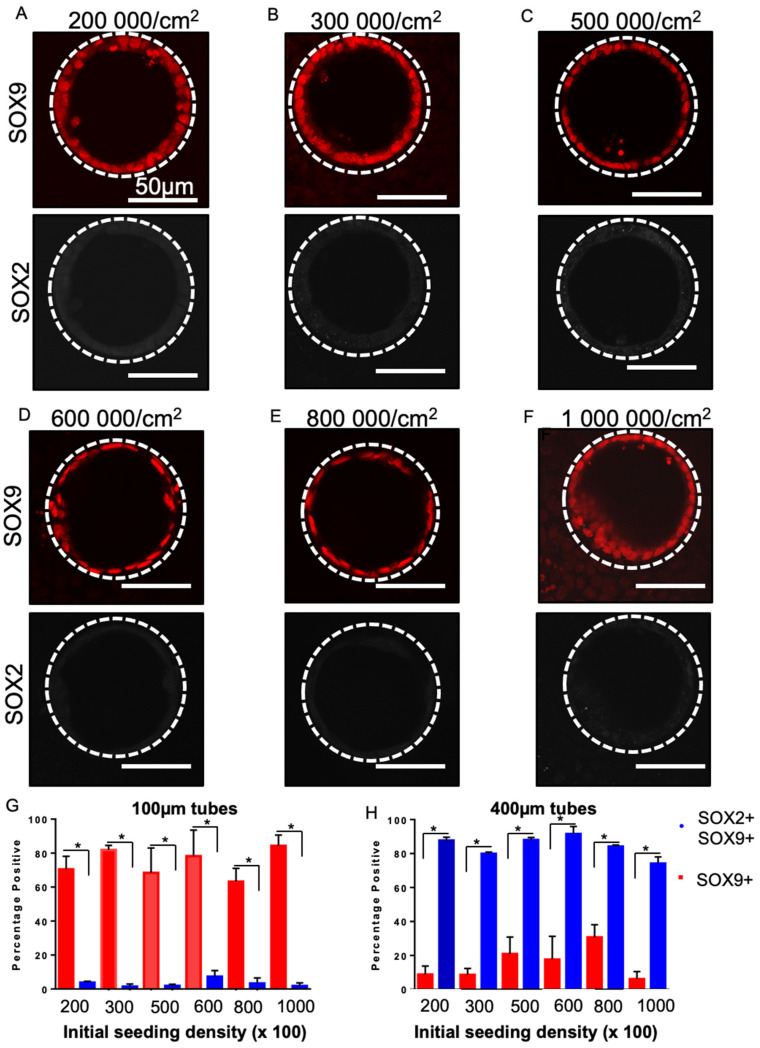
SOX2–SOX9 status for a range of different seeding densities. Fluorescent micrographs of day 17 lung progenitors seeded for 8 days in 100 µm tubes and stained for SOX9 (red) and SOX2 (white) are single positive for SOX9 in 200,000 cells/cm^2^ (**A**), 300,000 cells/cm^2^ (**B**), 500,000 cells/cm^2^ (**C**), 600,000 cells/cm^2^ (**D**), 800,000 cells/cm^2^ (**E**), 1,000,000 cells/cm^2^ (**F**). Bar charts showing quantification of the percentage of SOX9^+^ cells and the percentage of SOX9^+^SOX2^+^ cells in 100 µm tubes (**G**) and 1400 µm tubes (**H**) for a range of seeding densities. A significant increase in SOX9 single positive cells and a significant decrease in SOX2^+^SOX9^+^ was observed at all densities in 100-micron diameter tubes; * *p* < 0.05. Bar, 50 µm; the dotted white line approximates the PDMS border; n = 3.

Since our data suggested that cell geometry in the monolayer was sensitive to tube diameter but not initial seeding density (given a sufficient incubation time), we predicted that SOX2 status should also be insensitive to seeding density but dependent on tube diameter. As predicted, cells grown in 100 µm diameter tubes at all cell densities maintained SOX9 expression and lost SOX2 expression, resulting in a loss of double positive (SOX2^+^SOX9^+^) cells (Figure 6A–F). Conversely, in 400 µm diameter tubes at all densities, cells remained SOX2^+^SOX9^+^ (Supplementary Figure S4). Quantification (Figure 6G,H) revealed a consistent and significant difference between 100 and 400 µm tubes for both SOX9^+^ (Figure 6G) and SOX2^+^SOX9^+^ (Figure 6H) at all cell seeding densities (*p* < 0.05). For example, in the 600,000 cells/cm^2^ conditions in 100 µm tubes, 78.19 ± 15.34% cells were SOX9^+^ whereas in the 400 µm tubes cells were only 7.33 ± 3.51% SOX9^+^. Overall, this suggests that across a broad range of seeding densities cell self-organization is dictated by tube diameter, resulting in specific cell geometries that determine maintenance of SOX2 expression in the progenitor cells.

## 4. Discussion

Current directed differentiation protocols rely primarily on mimicking chemical cues that occur during development. There is evidence in the literature, however, supporting the use of biophysical cues to drive cell fate choice [36,37,38,39]. Indeed, standard directed differentiation cultures do show some spontaneous self-organization [6,40,41,42]; for example, lung buds [5] and renal tubules [41]. In these examples, however, organization is uncontrolled, heterogeneous, and does not recapitulate the final tissue organization and patterning observed in an embryo. Previous work in brain organoid cultures has also shown that introducing a synthetic physical cue can direct cell fate, the patterning of cell types present, and their physical organization, resulting in more recognizable tissue structures [43]. The use of artificially imposed biophysical cues therefore offers the opportunity to further improve the robustness of current directed differentiation protocols and guide the organization of the resulting differentiated cell populations. As a step towards this we previously showed that culturing lung progenitors in tubes with specific diameters, on the order of those observed in developing human embryos at the stage where early cell specification occurs [26], refines cell fate towards a distal lung lineage. Here we assessed the robustness of our seeding protocol to establish tube cultures and the impact of seeding parameters on inducing differentiation status changes.

Overall, our data suggest that self-organized monolayer formation is insensitive to initial cell seeding conditions (ECM composition, cell seeding density) if a sufficient incubation time (and hence number of cells) is used to confine the progenitor cells within the tube mould. This requirement for a critical number of cells to initially adhere within the tube to prevent death is likely dependent on cell type and differentiation status and influenced by cell-specific dependencies on cell–cell interactions. We speculate that monolayer formation is insensitive to ECM composition as long as sufficient cellular adhesions form to immobilize the cells in the tube, and that cells likely secrete their own matrix over time to facilitate monolayer formation over the tube surface. Furthermore, in our experiments, any chemical signals from the media were constant despite differences in initial ECM ligand activation on different ECM coatings. The observed insensitivity to ECM coating therefore potentially reflects the dominance of soluble signals in the system. Surprisingly, cell morphology within the self-organized monolayer was also insensitive to cell seeding density and depended only on initial tube diameter after appropriate washing. The tube surface therefore appeared to template the self-organized structure. We speculate that cell morphology in the self-organized structures is insensitive to initial seeding density because initial adhesion to the tube surface constrains the number of cells that are maintained in the tube to establish the monolayer.

Altering cell geometry has previously been shown to alter gene and protein expression in vitro [20,44] and in vivo [17]. For example, patterning melanoma cancer cell lines in particular geometries corresponding to shapes with lower radii of curvature increased the expression of specific cancer stem cell markers [44]. In another example, embryonic stem cells grown in microchambers of varying diameter (200, 400, and 600 μm) and stimulated towards a mesenchymal fate using WNT agonism successfully formed mesoderms in 400 and 600 µm diameter microchambers, as opposed to endoderms in 200 µm microchambers. Furthermore, cells grown within 400 and 600 µm microchambers created structures that mimicked the linear heart tube, expressing cardiac specific markers [45]. Both these examples highlight the potential of sub- and supra-cellular organization to induce specific alterations in cell differentiation status. Furthermore, in these cultures, the cell population was driven to more specific fates which reduced cell heterogeneity in the cultures. Our work further supports these two concepts—that geometric cues impose constraints on cells and thus homogenize cellular morphology and influence fate status.

Further work is necessary to fully dissect the molecular mechanism by which cell geometry translates into downstream changes in gene expression. Several specific biological mechanisms have emerged in model organisms [46] and mesenchymal stromal cells [47] as to how geometric cues could impact cell fate. In *C. elegans*, it was shown that Piezo 1 (a calcium channel) activity is altered based on external forces, such as stretch or compression. As Piezo 1 is stretched, calcium ions can more freely pass, signaling into the cell, which can alter the differentiation of gut cells [46]. Changes in membrane shape that result in ion-channel stretching or compression can therefore alter channel activation status and subsequent cell differentiation status. An alternative mechanism has been reported in mesenchymal stem cells (MSCs): when constrained within curvature, MSCs were shown to change in the ordering of the plasma membrane lipid bilayer, which in turn leads to alterations in AKT signaling that ultimately modulates MSC fate [46]. Both mechanisms provide a possible link between the physical organization of the cell membrane, which changes in different geometric configurations, and the internal signaling pathways that define cell fate, and are worth further exploration in the context of SOX2–SOX9 status in our system in the future. An additional area for future work would be to explore the specific diameter corresponding to loss of this architectural fate-guiding effect.

Given the robustness of our platform to induce tube self-organization and influence differentiation status, we speculate that this self-organization strategy is potentially scalable for incorporation into current standard differentiation and organoid protocols to drive specific fates and decrease culture heterogeneity. Beyond the lung, it would be interesting to see if other tubular-based organs, such as the pancreas, could also benefit from this biomimetic geometric culturing approach. Indeed, it is already known that NOTCH signaling, which drives pancreatic differentiation, is sensitive to changes in tensile forces applied to developing pancreatic progenitors [48]. Furthermore, pancreatic organoids have been shown to be sensitive to the number of cells within the initial aggregates, which presumably influences cell geometry among other things. Our tube culture approach could therefore provide a general method to control cell numbers within a defined architectural environment and potentially augment the control of directed differentiation protocols for a number of tissue types.

## 5. Conclusions

In conclusion, our study demonstrates that culture in tubes with specific diameter dimensions produces consistent changes in SOX status in ES-derived lung progenitors. This effect appears to be robust and unaffected by changes in ECM coating and cell seeding density. Interestingly, it appears that across a range of seeding densities cells seeded and washed appropriately will conform to the tubular diameter presented, resulting in monolayers of cells with similar maximum radii, perimeters, and areas. Our data support the concept of exploiting tissue-specific biophysical cues to improve directed differentiation by decreasing heterogeneity and enabling the creation of specific cell populations. Improving the robustness and predictability of directed differentiation cell manufacturing strategies is important for both developing in vitro tissues for drug development and for cell replacement. Beyond these applications, providing structural cues to induce appropriate self-organization of stem cells during differentiation is a potential strategy to create highly organized and patterned tissue structures that could serve as better models of development and disease than current organoid technologies which are unorganized and stochastically patterned. Ultimately, combining both arms of developmental biology via the use of both biophysical cues in combination with gold standard chemical-based protocols is likely necessary to induce the assembly of tissues with both correct composition and spatial organization.

## Data Availability

Not Applicable.

## Supplementary Materials

The following are available online at https://www.mdpi.com/article/10.3390/bioengineering8120209/s1, Figure S1: Advanced Biomatrix ECM Select Array Kit Ultra-36, extracellular matrix screening array setup. Figure S2: Cell seeding over a 48-hour time period. Figure S3: Cell seeding at various densities. Figure S4: SOX2, SOX9 status across different seeding densities.
