# Supplementary material for: Guided Self-Assembly of ES-Derived Lung Progenitors into Biomimetic Tube Structures That Impact Cell Differentiation
Source: Bioengineering (Basel). 2021 Dec 10;8(12):209. doi: 10.3390/bioengineering8120209 (PMC8698605; doi:10.3390/bioengineering8120209)
Supplement: Supplementary file 1 [file bioengineering-08-00209-s001.zip › bioengineering-1454539-supplementary.pdf]

Guided Self-Assembly of ES-derived Lung Progenitors into biomimetic tube structures that impact cell differentiation

Supplementary Information

	1	2	3
A	Collagen I (250µg/ml)	Collagen I (125µg/ml)<br>Collagen III (125µg/ml)	Fibronectin (125µg/ml)<br>Collagen IV (125µg/ml)
B	Collagen III (250µg/ml)	Collagen I (125µg/ml)<br>Collagen IV (125µg/ml)	Fibronectin (125µg/ml)<br>Collagen VI (125µg/ml)
C	Collagen IV (250µg/ml)	Collagen I (125µg/ml)<br>Collagen V (125µg/ml)	Fibronectin (125µg/ml)<br>Laminin (125µg/ml)
D	Collagen V (250µg/ml)	Collagen I (125µg/ml)<br>Collagen VI (125µg/ml)	Fibronectin (125µg/ml)<br>Vitronectin(125µg/ml)
E	Collagen VI (250µg/ml)	Collagen I (125µg/ml)<br>Fibronectin (125µg/ml)	Laminin (125µg/ml)<br>Collagen IV (125µg/ml)
F	Fibronectin (250µg/ml)	Collagen I (125µg/ml)<br>Laminin (125µg/ml)	Laminin (125µg/ml)<br>Collagen VI (125µg/ml)
G	Laminin (250µg/ml)	Collagen I (125µg/ml)<br>Vitronectin (125µg/ml)	Vitronectin (125µg/ml)<br>Collagen IV (125µg/ml)
H	Vitronectin (250µg/ml)	Collagen I (125µg/ml)<br>Topoelastin (125µg/ml)	Vitronectin (125µg/ml)<br>Collagen VI (125µg/ml)
I	Tropoelastin (250µg/ml)	Fibronectin (83.3µg/ml)<br>Laminin (83.3µg/ml)<br>Collagen I (83.3µg/ml)	Vitronectin (125µg/ml)<br>Laminin (125µg/ml)
J	Collagen IV (125µg/ml)<br>Collagen VI (125µg/ml)	Fibronectin (83.3µg/ml)<br>Laminin (83.3µg/ml)<br>Collagen IV (83.3µg/ml)	Vitronectin (125µg/ml)<br>Topoelastin (125µg/ml)
K	Collagen III (125µg/ml)<br>Collagen V (125µg/ml)	Vitronectin (62.5µg/ml)<br>Laminin (62.5µg/ml)<br>Collagen I (62.5µg/ml)<br>Collagen IV (62.5µg/ml)	Topoelastin (125µg/ml)<br>Collagen IV (125µg/ml)
L	Negative Control (250 µg/ml)	Fibronectin (62.5µg/ml)<br>Laminin (62.5µg/ml)<br>Collagen I (62.5µg/ml)<br>Collagen IV (62.5µg/ml)	Topoelastin (125µg/ml)<br>Collagen VI (125µg/ml)

Supplemental Figure S1. Advanced Biomatrix ECM Select® Array Kit Ultra-36, extracellular matrix screening array setup. From Advanced BioMatrix (https://www.advancedbiomatrix.com/extracellular-matrix-screening-array/ecm-select-array-kit-ultra-36/).

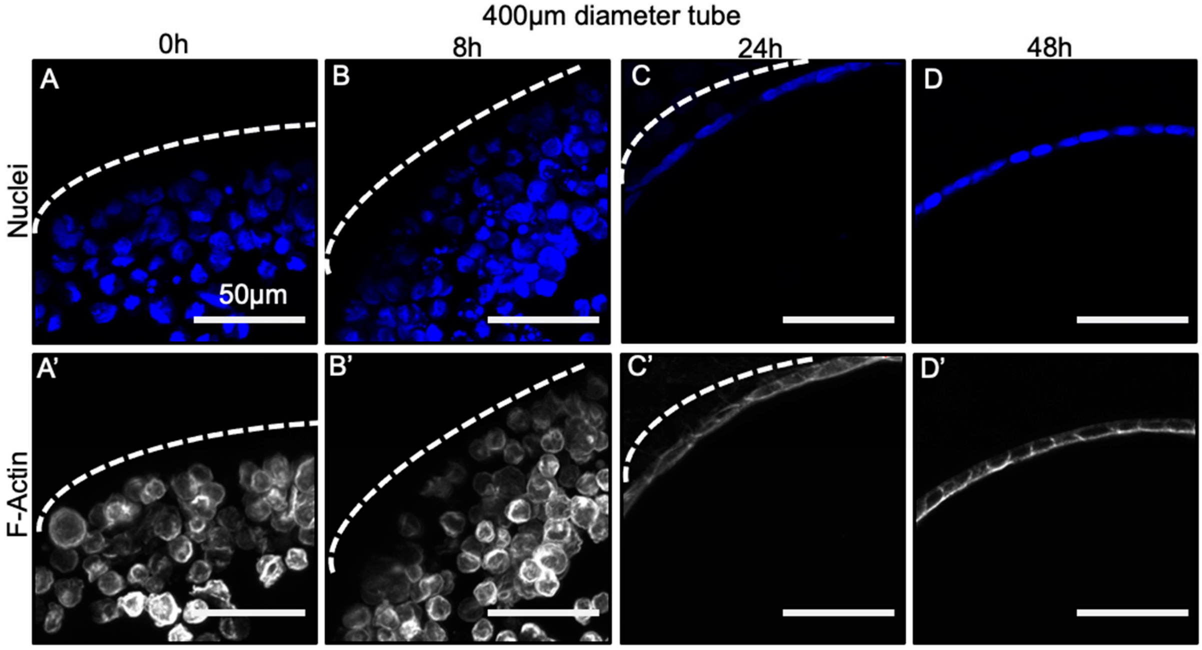

Supplemental Figure S2. Cell seeding over a 48 hour time period. Fluorescent micrographs of 400µm tubes seeded with day 17 lung progenitors and stained with DAPI (blue) at 0h (A), 8 hours (B), 24 hours (C), and 48 hours (D) showing partial tube formation by 8 hours and complete formation occurring within 48 hours. Corresponding F-actin staining (white) in (A'-D') reveals a similar pattern. Corresponding F-actin staining (white) in (E'-H') reveals a similar pattern; n=3.

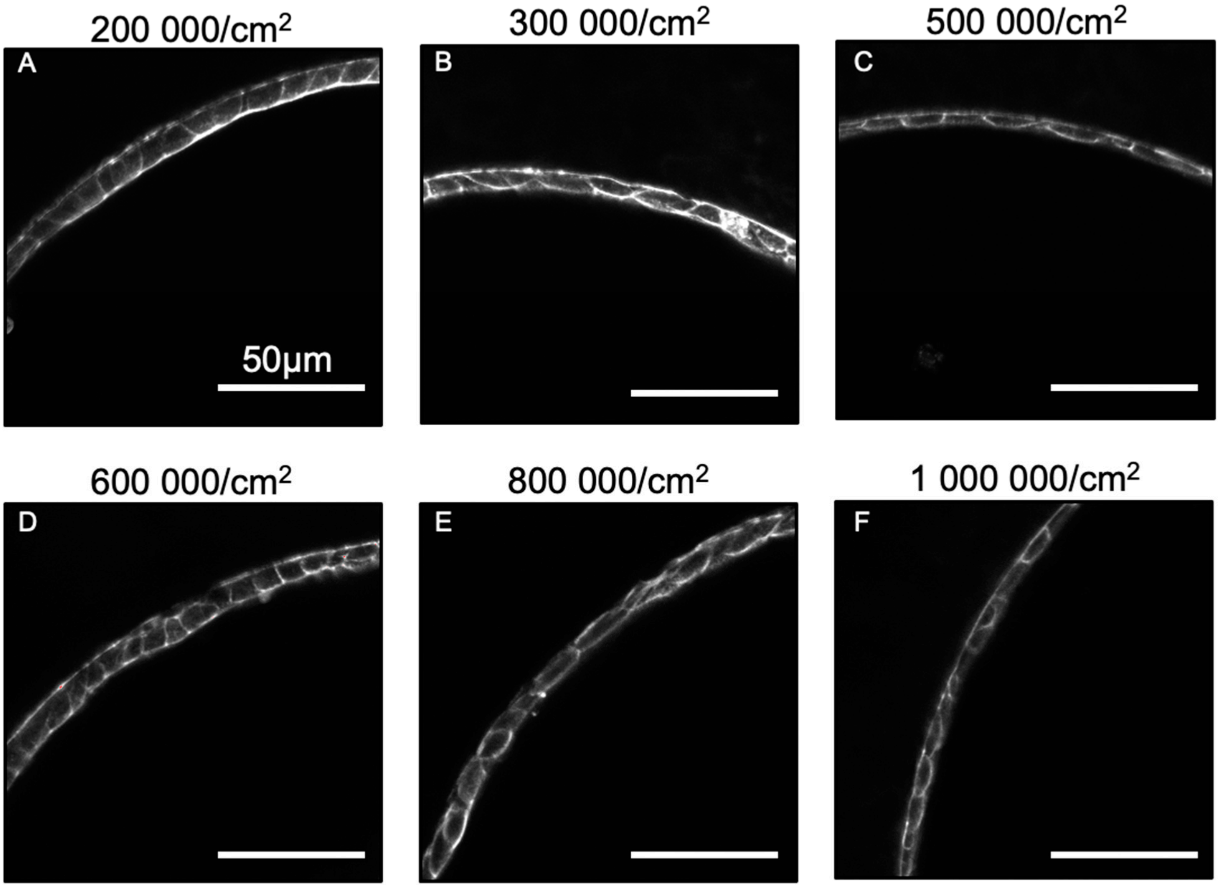

Supplemental Figure S3. Cell seeding at various densities. Fluorescent micrographs of day 17 lung progenitor cells seeded for 8 days in 400µm tubes and stained for F-actin (white) at 200 000 cells/cm^2 (A), 300 000cells/cm^2 (B), 500 000 cells/cm^2 (C), 600 000cells/cm^2 (D), 800 000 cells/cm^2 (E), 1 000 000cells/cm^2 (F) show that tube formation occurs without aggregate formation at these cell seeding densities; n=3.

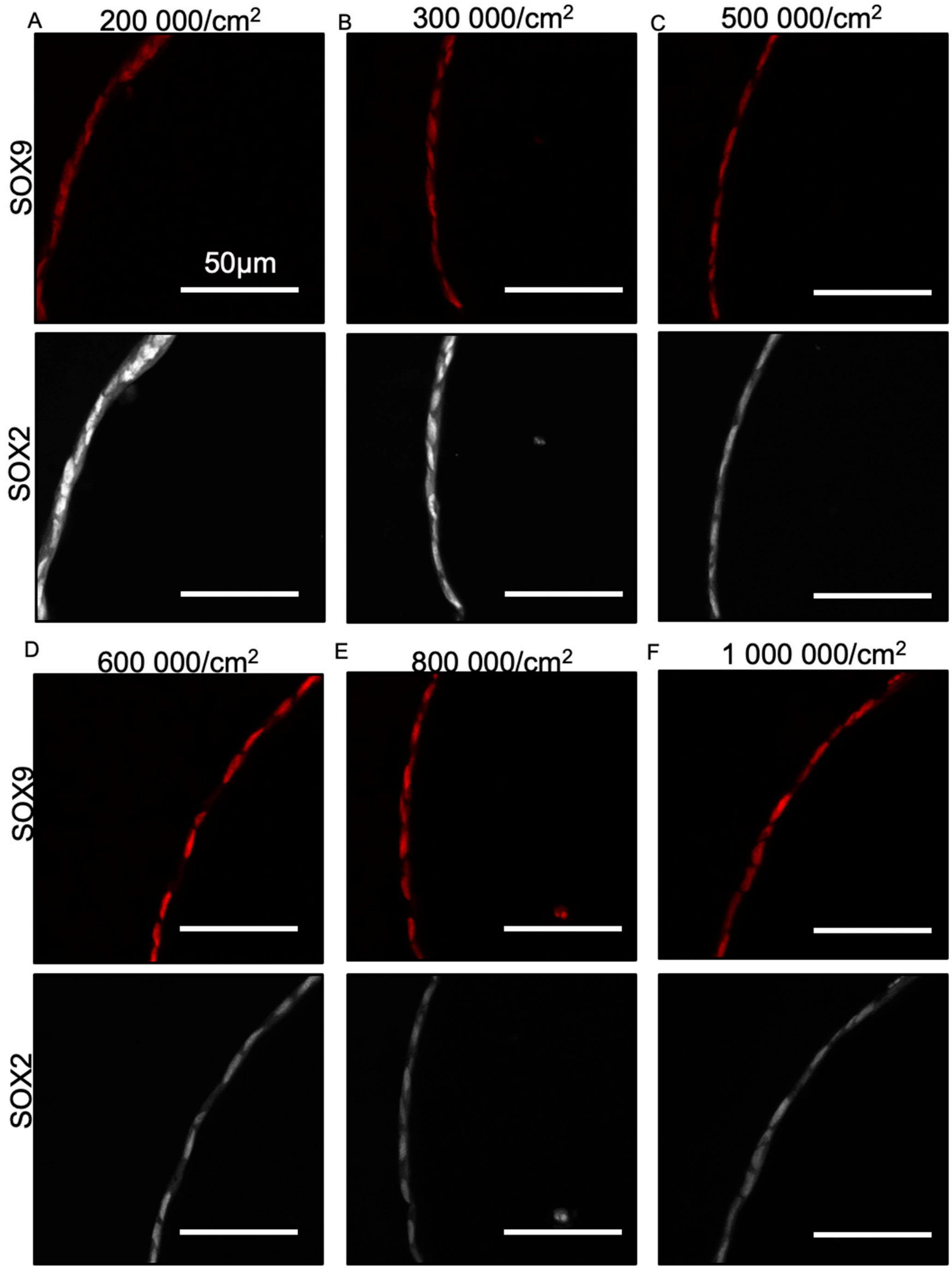

Supplemental Figure S4. SOX2, SOX9 status across different seeding densities. Fluorescent micrographs of day 17 lung progenitors seeded for 8 days in 400µm tubes and stained for SOX9 (red) and SOX2 (white) are single positive for SOX9 in 200 000 cells/cm^2 (A), 300 000 cells/cm^2 (B), 500 000 cells/cm^2 (C), 600 000 cells/cm^2 (D), 800 000 cells/cm^2 (E), 1 000 000 cells/cm^2 (F).
